# Identification of *BCL2L11* as a Candidate Gene for Hereditary Predisposition to Non‐Medullary Thyroid Cancer Using Familial Whole‐Exome‐Sequencing

**DOI:** 10.1111/cge.70060

**Published:** 2025-09-03

**Authors:** Duygu Abbasoglu, Mathis Lepage, Nicolas Sonnier, Sandrine Viala, Nancy Uhrhammer, Flora Ponelle‐Chachuat, Anne Cayre, Maud Privat, Mathias Cavaillé, Yannick Bidet

**Affiliations:** ^1^ Département d'Oncogénétique Centre Jean Perrin Clermont‐Ferrand France; ^2^ U1240 Imagerie Moléculaire et Stratégies Théranostiques Université Clermont Auvergne, INSERM Clermont Ferrand France; ^3^ Département de Pathologie Centre Jean Perrin Clermont‐Ferrand France

**Keywords:** *BCL2L11*, familial non‐medullary thyroid cancer, hereditary predisposition, *TELO2*, *UACA*, whole‐exome‐sequencing

## Abstract

Familial non‐medullary thyroid cancer, defined as two or more affected first‐degree relatives, accounts for 3%–9% of thyroid cancers. It is associated with more aggressive cancer, early age at diagnosis, multifocality, and increased risk of metastasis and recurrence. Although no high penetrance predisposing gene has been identified at present, the estimated contribution of genetics is significant. Our study explored five families presenting FNMTC using Whole‐Exome Sequencing and found three candidate genes: *TELO2* in one family, *UACA* and *BCL2L11* in another. All of these tumor suppressor genes are expressed in the thyroid, exhibit under‐expression in tumor tissue compared to healthy tissue both in silico and in our samples, and two of them are known to be involved in thyroid carcinogenesis via the FOXO3A pathway. Functional analysis to validate these candidate genes in thyroid cancer cells showed that one of the three, *BCL2L11*, has a tumor suppressor effect on proliferation and apoptosis. Their impact on hereditary predisposition to thyroid cancer, as well as their combined effects, requires further study. Indeed, a case–control study would be essential to determine the diagnostic utility of their routine analysis.

## Graphical abstract

Whole‐exome sequencing of five families with non‐medullary thyroid cancer revealed three candidate genes. Functional analyses confirmed *BCL2L11* as a strong candidate gene for hereditary predisposition to non‐medullary thyroid cancer.

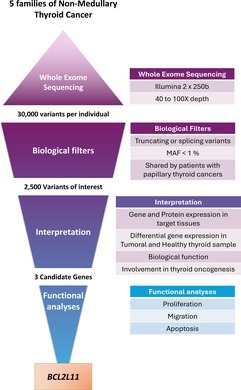

## 1 Introduction

Thyroid cancer is the most common endocrine malignancy, with an incidence of 821 214 cases worldwide in 2024 [1]. It includes medullary thyroid carcinoma (1%–2% of cases), derived from neuroendocrine C‐cells, and non‐medullary thyroid carcinoma (NMTC), derived from follicular epithelial cells that represent 95% of thyroid cancers. Papillary thyroid carcinoma (PTC) is the most common of these, accounting for 90% of cases [2]. PTC is associated with a lifetime risk estimated at 1.1% in the general population, with female predominance and median age at diagnosis of about 50 years [3].

The main risk factors of NMTC include age, gender, goitre, and irradiation, particularly during childhood or adolescence [4, 5]. Furthermore, NMTC is included in certain rare syndromes of hereditary predisposition to cancer, including PTEN Hamartoma syndrome, familial polyposis syndrome, Werner syndrome, DICER1 syndrome, and Carney complex [6, 7]. However, NMTC is secondary in these syndromes.

Persons with a first‐degree relative with PTC have an increased risk of thyroid cancer, suggesting the presence of genetic factors, in particular in familial NMTC (FNMTC) [8]. FNMTC, defined as two or more affected first‐degree relatives, represents 3%–9% of thyroid cancers [7]. The contribution of genetic factors is estimated at > 50% when two first‐degree relatives are affected and at > 95% when three members are affected [9]. This form of cancer is associated with more aggressive tumors, early age at diagnosis, multifocality, and increased risk of metastasis and recurrence. Furthermore, thyroid cancer patients have an increased risk of second cancers of different types [10]. Thus, the identification of FNMTC is important to provide these families with appropriate medical care, and the identification of predisposition genes would help to improve this care.

Some susceptibility loci have been identified, including *TCO*, *PRN*, *NMTC1*, *MNG1*, *PTCSC3*, and *FTEN*. However, no high penetrance gene has yet been identified [7, 11]. Pangenomic analyses have shown that FNMTC is genetically heterogeneous, preventing the discovery of strong candidate genes using association studies. Conversely, individual pedigrees considered separately could lead to interesting insights [12]. Furthermore, the detection of variants is only the first step in identifying a candidate gene, whose causative role needs to be supported by functional analyses.

Our study used Whole Exome Sequencing of severe cases of FNMTC with > 2 affected first‐degree relatives to identify high penetrance candidate genes.

## 2 Patients, Materials and Methods

### 2.1 Samples

**FIGURE 1:**
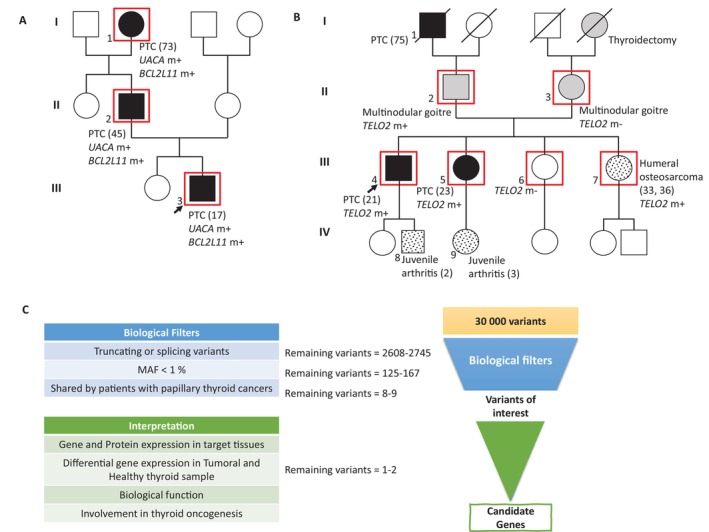
Selection criteria of candidate genes. The number between brackets corresponds to the age of occurrence of the disease. Individuals represented by a black form were diagnosed for Papillary Thyroid Carcinoma (PTC). Grey signs represent individuals with non‐tumoral thyroid disease. Speckled forms report individuals with unrelated disease. Red squares indicate sequenced patients. The “m” following the gene name signals if the individual is mutated (+) or not (−). The proband is indicated by an arrow. (A) Family A presents familial non‐medullary thyroid carcinoma, with 3 related cases at first degree pairwise with a PTC, over 3 successive generations. The suspected mode of transmission is autosomal dominant. There is also a phenomenon of anticipation, with the occurrence of thyroidal cancer growing younger over the generations. (B) Family B presents a familial non‐medullary thyroid carcinoma, with 3 related cases in the same parental branch with a PTC. The suspected mode of transmission is autosomal dominant. Each parental branch may be considered at risk because of the occurrence of multinodular goitre in the mother and father. (C) Biological Filters focus on rare, truncating variants shared by patients. Individual interpretation of genes of interest includes expression in tumoral and healthy tissues, as well as known biological function in thyroid. MAF: minor allele frequency.

Five highly evocative families of hereditary non‐medullary thyroid carcinoma were included from the oncogenetic consultation of the Centre Jean Perrin. Family eligibility criteria included at least two cases of thyroid cancer in first‐degree relatives, the availability of DNA from at least two thyroid cancer patients in the family, the presence of tumour blocks from thyroid cancer available for at least one family member, and the absence of a known variant in the APC, PTEN, and WRN predisposition genes. A diagnostic analysis revealed no pathogenic mutation in a panel of cancer predisposition genes (Table 1). After giving informed consent for genetic diagnosis and hereditary disease research, three individuals from Family A, five from Family B, three from Family C, four from Family D, and three from Family E were analysed further (Figure 1, Supplemental Data). The study is registered under number 2020/ce 58 with the local ethics committee.

**TABLE 1:** Diagnostic gene panel.

Panel of diagnosis genes sequenced			
**APC**	(LRG_130t1 and t2)	**NF2**	(LRG_511 t1)
**ATM**	(LRG_135)	**PALB2**	(LRG_308)
**BAP1**	(LRG_529)	**PMS2**	(LRG_161)
**BMPR1A**	(LRG_298)	**POLD1**	(LRG_785 t1)
**BRCA1**	(LRG_292)	**POLE**	(LRG_789)
**BRCA2**	(LRG_293)	**PTEN**	(LRG_311)
**BRIP1**	(LRG_300)	**RAD51C**	(LRG_314)
**CDH1**	(LRG_301)	**RAD51D**	(LRG_516)
**CDKN2A**	(LRG_11t1 & t2)	**RET**	(LRG_518 t1)
**CHEK2**	(NM_007194.3)	**SDHA**	(NM_004168.3)
**EPCAM**	(LRG_215)	**SDHAF2**	(LRG_519)
**FLCN**	(LRG_325)	**SDHB**	(LRG_316)
**MAX**	(LRG_530)	**SDHC**	(LRG_317)
**MEN1**	(LRG_509 t2)	**SDHD**	(NM_003002.3)
**MLH1**	(LRG_216)	**SMAD4**	(LRG_318)
**MSH2**	(LRG_218)	**STK11**	(LRG_319)
**MSH6**	(LRG_219)	**TMEM127**	(LRG_528)
**MUTYH**	(NM_001048171.1)	**TP53**	(LRG_321 t1)
**NBN**	(LRG_158)	**VHL**	(LRG_322)

*Note*: Thirty‐eight genes involved in predisposition to cancer, including PTC, were analysed in all patients included in our study. DICER1 and PRKAR1A were not included in this panel. No patient in our study presented a probably pathogenic or pathogenic variant in genes analysed.

### 2.2 Clinical Presentation of the Selected Families With FNMTC

Family A (Figure 1A): The proband, at 17 years of age, had a 50 mm unilateral papillary thyroid carcinoma in the right lobe with latero‐cervical and mediastinal lymph node invasion (T3N1bM0). His father presented at age 45 with bilateral multifocal PTC (21 mm) and similar lymph node invasion (T1N1bM0), plus multiple thyroid nodules. The paternal grandmother had bilateral multifocal PTC (25 mm) with vascular and peri‐thyroid adipose tissue invasion but no lymph node involvement (T4N0M0).

Family B (Figure 1B): The proband presented at age 21 with a unilateral, unifocal PTC of the left lobe, 5 mm in diameter (T1N0M0). His sister presented a bilateral multifocal PTC 20 mm in diameter at age 23 (T2N0M0). Another sister presented a left humeral osteosarcoma at ages 33 and 36 of age. An additional sibling had no cancer. Both parents presented multinodular goitres. The paternal grandfather (75) presented a PTC, and the maternal grandmother had a thyroidectomy for an unknown cause. Juvenile arthritis was present in 2/5 of the children of the fourth generation.

Family C (Supplemental Data): The proband presented at age 49 with a subcapsular PTC of the left lobe measuring 11 mm in the long axis (T1bN1M0). Her two daughters had PTC at the ages of 26 and 29, with a 25 mm nodule corresponding to a papillary carcinoma of the right lobe (T2N0M0) and a 6 mm subcapsular papillary adenocarcinoma of the right lobe, respectively (T1N0M0). The proband's sister presented with goitre, and one of her aunts underwent thyroidectomy for a benign pathology. One of her cousins presented with multiple thyroid nodules, and two other cousins presented with hypothyroidism.

Family D (Supplemental Data): The proband presented with PTC at age 52 (T1N0M0). Her brother had the same diagnosis (T1N0M0) at age 55. Her son had an operation for a thyroid tract cyst‐thyroglossus 35–15 mm with an osseous fragment 15–10 mm. This family was included because, although there were only two cases of thyroid cancer, two other brothers had thyroid nodules, one of whom had early surgery (age 28), and we had the possibility of analyzing all family members with malignant and benign thyroid disease.

Family E (Supplemental Data): The proband was diagnosed with an 8 mm right thyroid microcarcinoma (T1N0M0) at age 37 and breast cancer at 43. Her sister had a 2 mm right thyroid microcarcinoma (T1N0M0) with multinodular goitre. Their maternal aunt and two of her maternal aunt's daughters also had PTC. This large 5‐generation family (55 females, 54 males) shows hereditary cancer: 13 with thyroid issues (5 thyroid cancers, at least 6 with multiple nodules) and 5 females with breast cancer. The simplified pedigree is in the Supplemental Data.

### 2.3 Germline Whole Exome Analyses

#### 2.3.1 Sequencing

DNA was extracted from peripheral blood using QIAamp DNA maxi‐kit (Qiagen) following the manufacturer's guidelines. Sonic fragmentation was performed on a Bioruptor instrument (Diagenode). Kapa library preparation kit and SeqCap EZ MedExome kit (Roche) were used for library preparation and capture. Sequencing was performed using a NextSeq 500/550 High Output v2 kit (300 cycles) on a NextSeq 500 instrument (Illumina). All steps were performed following the manufacturer's guidelines.

#### 2.3.2 Bio‐Informatic Analysis

For families A to D, de‐multiplexing was performed using bcl2fastq2 Conversion Software (Illumina). Alignments were performed on the Genome Reference Consortium Human Genome Build 37 (GRCh37 hg19) using Burrows‐Wheeler Aligner (v0.7.12‐r1039). Genome Analysis Toolkit 3.5 (GATK) and PICARD 1.129 tools were used for base quality score recalibration, realignment (and PCR duplicates removal, as recommended by Eurogentest guidelines) [13]. Average depth was calculated according to targeted regions defined in the bed file provided by Roche. Variant calling was performed using GATK HaplotypeCaller based on the GATK best practice protocol and annotated using Ensembl VariantEffectPredictor (v91) and Annovar (v17‐07‐2017). Variants were filtered for quality score ≥ 30, depth ≥ 20×, and present in ≥ 20% of reads. Analysis of copy number variations was only performed for genes included in the diagnostic panel (Table 1). Due to inclusions spanning a long period of time, Family E was analyzed according to the same pipeline but newer versions of some tools (genome version GRCh38, GATK 4.2.6, Picard 2.26, VEP 109.3, Annovar 2020‐06‐08).

#### 2.3.3 Biological Filters

To identify variants of interest involved in monogenic hereditary cancer predisposition with high penetrance, biological filters included: minor allele frequency < 1% or unknown to gnomAD, truncating variants (nonsense, frameshift), and splice‐site variants with significant MaxEntScan score difference above 30%. Except for those with an impact on splicing, synonymous and missense variants were not considered (Figure 1C).

#### 2.3.4 In Silico Analysis

Each variant of interest was annotated and interpreted with the aid of Alamut Visual 2.11 (Interactive BioSoftware), which includes splice site analysis tools (MaxEntScan) and protein function prediction tools (SIFT, Polyphen 2.0). Genes carrying variants of interest presenting RNA and protein expression in the thyroid tissue were selected, using the protein atlas database (proteinatlas.org). The function of each gene of interest was interpreted using Genecards (genecards.org), Uniprot (uniprot.org) and Pubmed (https://pubmed.ncbi.nlm.nih.gov/). Each variant was confirmed by direct reading of sequence using Integrative Genomics Viewer (IGV, Broad Institute: https://software.broadinstitute.org/). Proposed splice site variants were validated using SPiP (v1.1, threshold > 0.7) [14] and SpliceAI software (> 0.2).

Differential gene expression between tumor and healthy thyroid samples was explored using GEPIA (Gene Expression Profiling Interactive Analysis) and Expression Atlas databases (Figure 1C).

### 2.4 Somatic Analyses

#### 2.4.1 Loss of Heterozygosity Analysis

DNA was extracted from formalin‐fixed paraffin‐embedded thyroid samples, using Maxwell 16 FFPE Plus LEV kit. Primers were designed using PrimerBlast to target germline variants identified by WES. Primers and experimental conditions are available on request. Loss of heterozygosity (LOH) was studied by Sanger dideoxy sequencing for these same variants in the patients' thyroid tumor samples. LOH was defined by an 80% reduction of an allele, using Seqman software v12 (DNASTAR).

#### 2.4.2 Protein Expression Analysis

Protein expression in healthy and tumour thyroid samples was analyzed using Thermo Fisher antibodies: monoclonal BCL2L11 (MA5‐14848), and polyclonal TELO2 (PA5‐59583) and UACA (PA5‐50883). Dilutions were 1:100 for BCL2L11 and TELO2, and 1:50 for UACA. BCL2L11 targets Proline‐25, present in all isoforms. Samples underwent CC1 pre‐treatment (Roche Ventana, 60 min at 95°C), followed by 1 h antibody incubation at 37°C. Detection used the VENTANA UltraView DAB system.

### 2.5 Functional Analyses

#### 2.5.1 Cell Culture

Two human thyroid cancer lines (TPC1 and 8305‐C) were chosen to be the closest to the papillary tumors developed by the patients. The choice also considered the basal expression of the genes to be analyzed, determined from the CCLE database (Broad Institute Cancer Cell Line Encyclopedia). They are cultured in RPMI‐1640 and DMEM media, respectively, as recommended by the supplier. 8305‐C was purchased from the ATCC, and TPC1 was obtained from the U1195 Inserm platform.

#### 2.5.2 siRNA Experiments

The siRNAs used consist of a mixture of siRNAs directed against each gene (ON‐TARGET plus Smart Pool, BCL2L11: L‐004383‐00‐0010, UACA: L‐013795‐00‐0010, TELO2: L‐021207‐01‐0010, and non‐targeting control: D‐001810‐10‐50; Horizon Discovery). Transfections were performed using Lipofectamine RNAiMAX (Life Technologies) according to the manufacturer's instructions. TPC1 and 8305C cells were seeded at 2.5 × 10^5^ and 3.5 × 10^5^ cells/well in 6‐well plates, and transfected 24 h later with 10 nM siRNA.

#### 2.5.3 RT‐qPCR

Forty‐eight hours post‐transfection, RNA was extracted and quantified using a Qubit 4 Fluorometer (Thermo Fisher). cDNA synthesis was performed with SuperScript III (Life Technologies), followed by qRT‐PCR to assess gene expression relative to the housekeeping gene PPIA, selected for its low expression variation between healthy and tumor thyroid tissues. qRT‐PCR was run on the QuantStudio 5 System (Thermo Fisher, A43183) and analyzed with Design and Analysis 2 software (v2.6). Inhibition rates were calculated by comparing RNA levels of the target gene in cells transfected either with siRNA directed against target genes or with a control siRNA that does not target any human gene.

#### 2.5.4 Analysis of Protein Levels Expression by Western Blotting

Forty‐eight hours after transfection, cells were lysed in RIPA buffer (Thermo Fisher) with protease and phosphatase inhibitors. Protein concentration was measured using the Pierce BCA assay. For western blotting, proteins were separated via SDS‐PAGE (Bio‐Rad TGX 4%–15% gels) and transferred to PVDF membranes. Membranes were blocked with 5% BSA (1 h, RT), then incubated overnight at 4°C with primary antibodies: Anti‐UACA (Progen, 691 535), Anti‐BIM (Invitrogen, MA5‐14848), Anti‐TELO2 (ABClonal, A17064), and Anti‐GAPDH (Cell Signaling, #5174). Secondary antibodies (Calbiochem Goat Anti‐mouse IgM JA 1200 and Cell Signaling Anti‐rabbit IgG HRP 7074P2) were used at a 1:2000 dilution, and protein bands were detected by enhanced chemiluminescence (ECL Prime, Amersham RPN2236) following the manufacturer's protocol.

#### 2.5.5 Apoptosis Studies

Two days after siRNA transfection, TPC1 and 8305‐C cells were subjected to cellular stress with Cisplatin (30 and 25 μM respectively) for 48 h. The cells were then labeled with propidium iodide (PI) and FITC‐annexin V (Annexin V Apoptosis Detection Kit FITC (Thermo Fisher)) and revealed by BD LSR II flow cytometer (Becton‐Dickinson). The number of living cells was taken as a basis when evaluating the results.

#### 2.5.6 Proliferation and Migration Studies

After transfection, the cells were incubated in an image acquisition device (Incucyte from Sartorius AG) to monitor the evolution of confluence. The acquisition of the images was carried out automatically every 2 h for at least 72 h. Proliferation analysis was performed for both cell lines until full confluence was reached, which occurred at 26 h for TPC1 and 48 h for 8305. The experiment was repeated 4 times for each cell line. Proliferation results were measured using Incucyte 2022 Rev1.

## 3 Results

### 3.1 Whole Exome Sequencing

Prior to WES, a diagnostic panel of 38 genes involved in predisposition to cancer was analyzed for all patients. None of them present a pathogenic or probably pathogenic variant in these genes (Table 1). As *DICER1* and *PRKAR1A* were not included in this panel, the first step of our WES analysis was to search for variants specifically in these genes, and none was found.

#### 3.1.1 A—WES Of Family A

WES of Family A presented an average depth of 84.7 reads (X) on targeted regions. The three persons analyzed presented an average of 30 011 variants meeting quality criteria, with an average of 2608 truncating and splice‐site variants (Figure 1C). After frequency and bio‐informatic filtering, eight truncating and splice‐site variants were shared by the three patients (Table 2). Two variants of interest were identified in two genes suspected of being tumor suppressor genes, as genes are expressed in thyroid tissue and both their mRNA and protein are underexpressed in PTC: a *BCL2L11* splice site variant NM_138621.4:c.498 + 3A>T; p.? and a frameshift variant NM_018003.3:c.3701del; p.(Ser1234Metfs*10) in *UACA*. SpIP and Splice AI predict that the c.498 + 3A>T variant of *BCL2L11* highly affects the donor splice site of exon 3 (SpIP: Alteration of the consensus splice site 98.41% [91.47%–99.96%]; SpliceAI: DL:0.91 (−3)).

**TABLE 2:** Variants of interest in family A (A) and family B (B).

Table 2A								
Gene	Variant	NP	I‐1	II‐2	III‐3	Expression in target tissues	GEPIA	Biological function
BCL2L11 (NM_138621.4)	**c.498 + 3A>T**	**p?**	**1**	**1**	**1**	**y**		**Pro‐apoptotic protein/TGS**
CCDC178 (NM_001308126.1)	c.457 + 2 T>C	p?	1	1	1	n	NA	Coiled‐coil domain‐containing protein
COL27A1 (NM_032888.3)	c.3716G>A	p?	1	1	1	n	NA	Collagen
FAM166B (NM_001164310.2)	c.745_748dup	p.Phe250*	1	1	1	n	NA	Protein FAM
GJA10 (NM_032602.1)	c.1477C>T	p.Gln493*	1	1	1	n	NA	Gap junction protein
PAMR1 (NM_015430.3)	c.397C>T	p.Arg133*	1	1	1	y	N	Ca + ion binding protein
RESP18 (NM_001007089.3)	c.508G>T	p.Glu170*	1	1	1	n	NA	Cortisol regulation protein
SERGEF (NM_012139.3)	c.285C>A	p.Cys95*	1	1	1	y	N	GEF
UACA (NM_018003.3)	**c.3701del**	p.Ser1234Metfs*10	**1**	**1**	**1**	**y**	**N**	**Pro‐apoptotic protein/TGS**

Table 2B											
Gene	Variant	NP	III‐4	III‐6	III‐7	II‐3	II‐2	III‐5	Expression in target tissues	GEPIA	Biological function
CILP (NM_003613.3)	c.2935C>T	p.(Gln979*)	x	x	0	0	x	x	n	N	Cartilage scaffolding
ENGASE (NM_001042573.2)	c.471C>A	p.Cys157*	x	x	x	0	x	0	y		Glycosidase
LAP3 (NM_015907.2)	c.1260 + 5G>T	p.?	x	x	0	0	x	x	y	N	Aminopetitdase
MAP3K6 (NM_004672.4)	c.1256‐2A>G	P?	x	x	x	x	0	0	y		Pro‐apoptotic protein
MICALCL (NM_032867.3)	c.885G>A	p.(Trp295*)	x	x	0	0	x	x	y	N	Protein kinase
OR2AP1 (NM_001258285.1)	c.93del	p.Ala32Argfs*10	x	x	x	x	0	0	n	NA	Olfactory receptor
SPOCK2 (NM_014767.2)	c.1058del	p.(Gly353Valfs*13)	x	x	0	x	0	0	y		Extracellular matrix protein
**TELO2 (NM_016111.3)**	**c.1792C>T**	p.Gln598*	**x**	**x**	**x**	**0**	**x**	**0**	**y**		**Regulator protein kinase**
TLDC1 (NM_020947.3)	c.43_44del	p.(Gln15Valfs*4)	x	x	x	x	0	x	y	N	mTOR activator
UBE2D3 (NM_181893.2)	c.17del	p.Lys6Serfs*49	x	x	x	0	x	0	y	N	Ubiquitinase

*Note*: Variants of interest were defined as rare (MAF < 1%) truncating variants shared by patients with PTC. Expression target tissues include mRNA and/or protein expression in thyroid using proteinatlas database (proteinatlas.org). GEPIA analysis determines gene expression in tumor tissue compared to corresponding healthy tissue.

Abbreviations: 0: absent; GEF: guanine exchange factor; n: No; NA: Not applicable.; NP: nomenclature protein; TSG: tumor gene suppressor; x: present; y: yes.

#### 3.1.2 B—WES Of Family B

WES of Family B presented an average depth of 98×. All six persons analyzed presented an average of 30 653 variants meeting quality criteria, with an average of 2745 truncating variants (Figure 1C). After frequency and bioinformatics filtering, nine truncating and splice‐site variants were identified (Table 2). One variant of particular interest was identified: the nonsense variant NM_016111.3:c.1792C>T; p.Gln598* in TELO2. This variant, inherited from the father, was identified in his two children with thyroid cancer and in his daughter with osteosarcoma.

#### 3.1.3 C—WES Of Other Families

For families C, D, and E, the average depth of WES was 41.6, 40.8, and 57.1, respectively. These families revealed 28 542, 28 213, and 29 070 variants on average, including 2528, 2417, and truncating variants, respectively. Although the sequencing quality and numbers were similar to the first two families, no gene was selected after the interpretation of the variants according to Figure 1C.

### 3.2 Sanger Sequencing Analysis

For Family A, a thyroid cancer sample from patient 1 was available for analysis. Sanger sequencing of the tumor confirmed the variants of interest in *UACA* and *BCL2L11* in a second independent sample, with no LOH observed (data not shown). For Family B, thyroid cancer samples from patients III‐4 and III‐5 were available for analysis. Sanger sequencing confirmed the variant of interest in *TELO2*, with no LOH observed (data not shown).

### 3.3 Immunohistological Analyses

**FIGURE 2:**
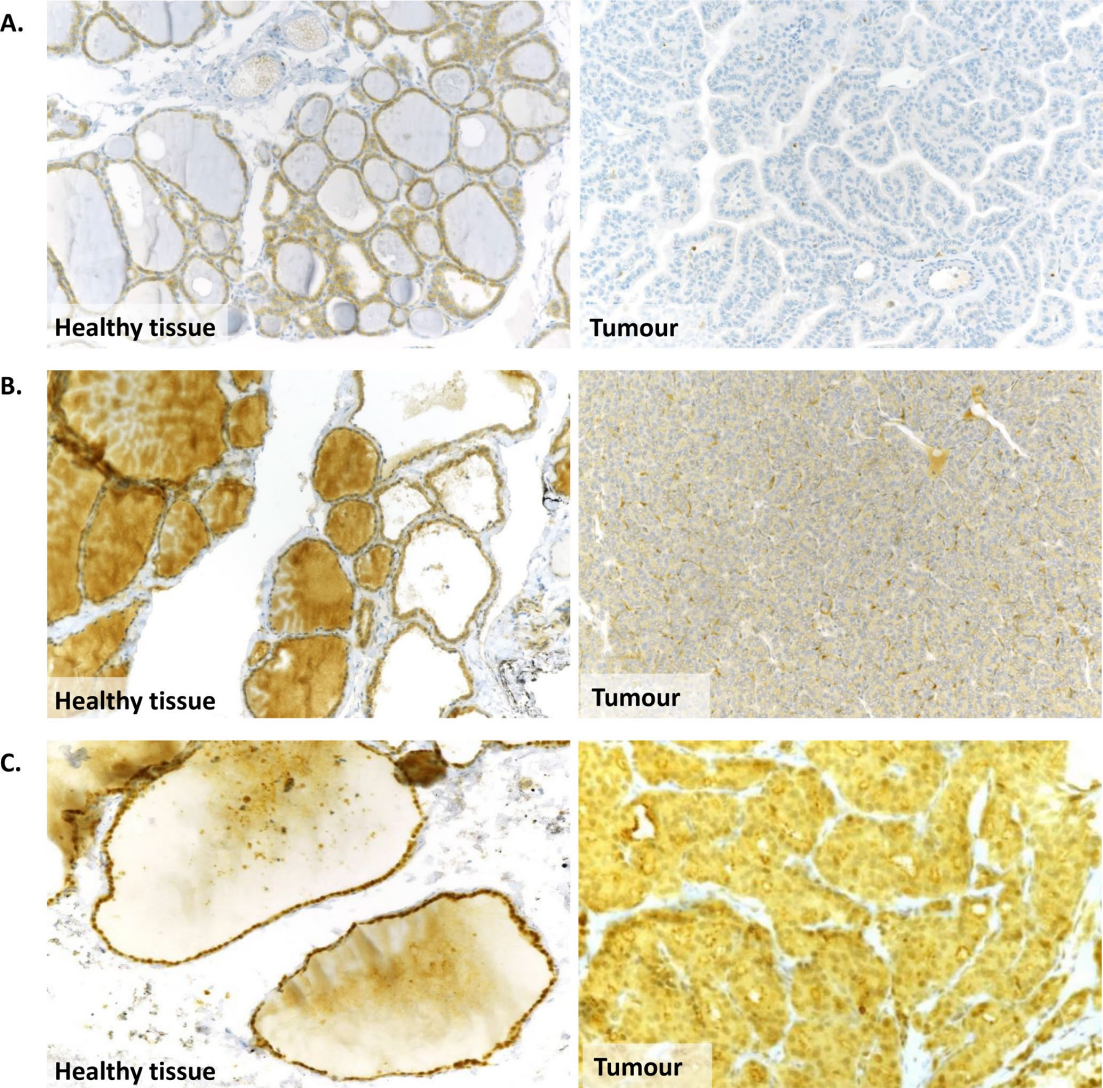
Protein accumulation in healthy and corresponding thyroid tumour samples. A complete loss of protein staining is observed in tumour samples in comparison to corresponding healthy samples. This confirms the deleterious impact of the variants, and this complete absence of protein suggests a second hit in favor of an involvement in thyroid oncogenesis. (A) BCL2L11 staining in patient A‐III.3 (×10), healthy and thyroid tumour samples. (B) UACA staining in patient A‐III.3 (×10), healthy and thyroid tumour samples. (C) TELO2 staining in patient B‐III.4 (×20), healthy and thyroid tumour samples. Patient B‐III.5 shows a similar loss of staining in tumour.

IHC showed a complete loss of expression of BCL2L11 and UACA in the thyroid tumour sample of patient III‐3, Family A (Figure 2A,B). In Family B, underexpression of TELO2 was observed in the nuclei of the thyroid tumour samples of patients III‐4 and III‐5 (Figure 2C).

### 3.4 Inhibition of Candidate Genes

**FIGURE 3:**
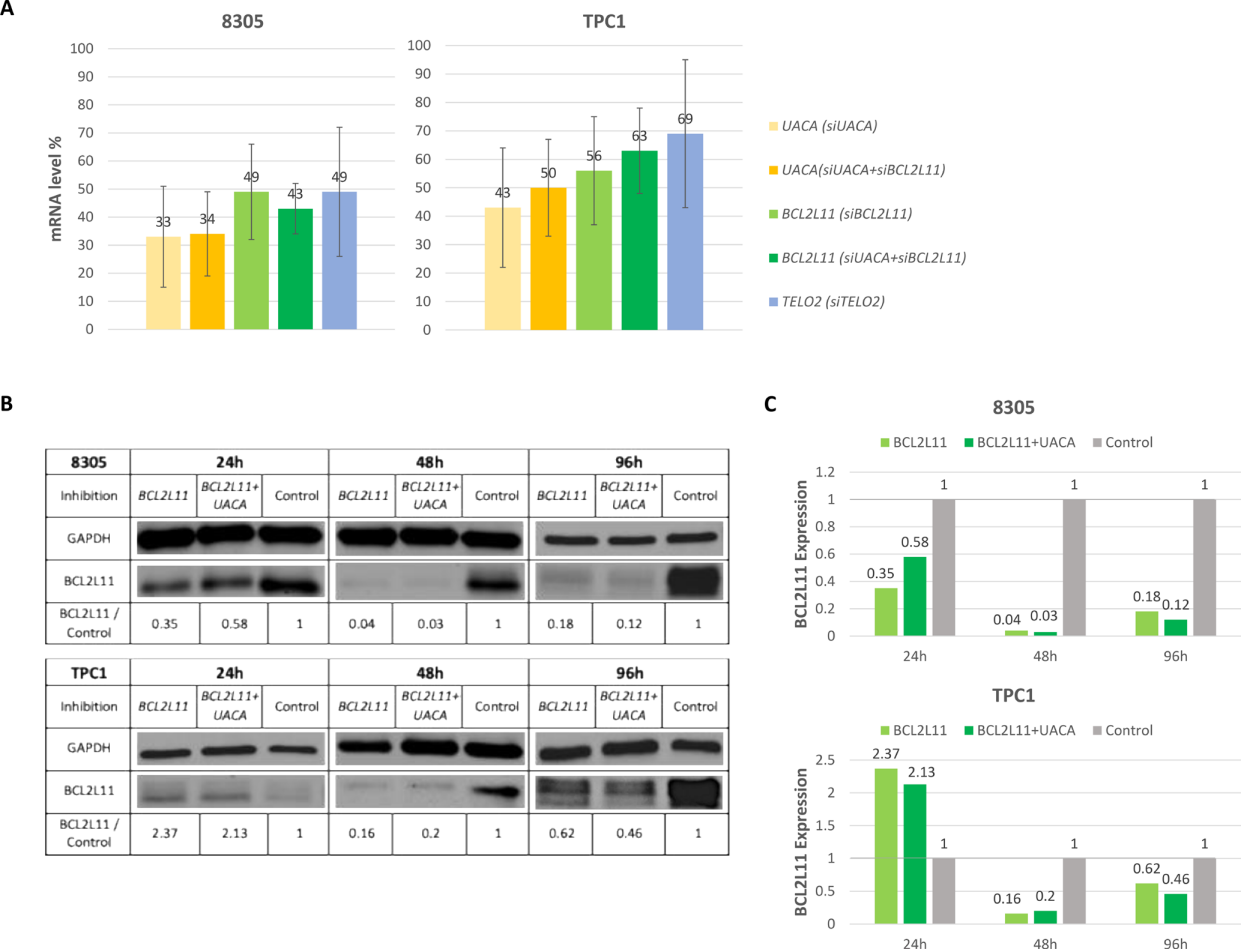
RNA and protein inhibition of *UACA*, *BCL2L11*, *and TELO2*. UACA and BCL2L11 siRNAs were used alone or in combination to mimic the presence of 2 mutations in Family A. RNA interference was performed on 8305 and TPC1 thyroid cancer cell lines. (A) Inhibition rates of *UACA*, *BCL2L11* and *TELO2*, measured by RT‐qPCR after specific siRNA treatment. (B) Western blot analysis of BCL2L11 in 8305 and TPC1 cells after single or combined BCL2L11‐UACA inhibition. Proteins were extracted at 24, 48, and 96 h post‐siRNA transfection. (C) Inhibition rates for BCL2L11 protein according to time after transfection: The most effective protein decrease was observed after 48 h for both cell lines.

To confirm the link between the candidate genes and FNTMC, the genes were knocked down in thyroid cancer cell lines via siRNA, and the level of inhibition was measured by RT‐PCR. The 8305 cell line exhibited higher inhibition rates than the TPC1 cell line (Figure 3A). Western blot analysis revealed a delayed decrease in BCL2L11 protein levels from day 2 post‐transfection, which persisted until at least day 4 (Figure 3B). UACA and TELO2 proteins could not be reliably quantified.

### 3.5 *UACA* , *BCL2L11* , and *TELO2* Effect on Proliferation

**FIGURE 4:**
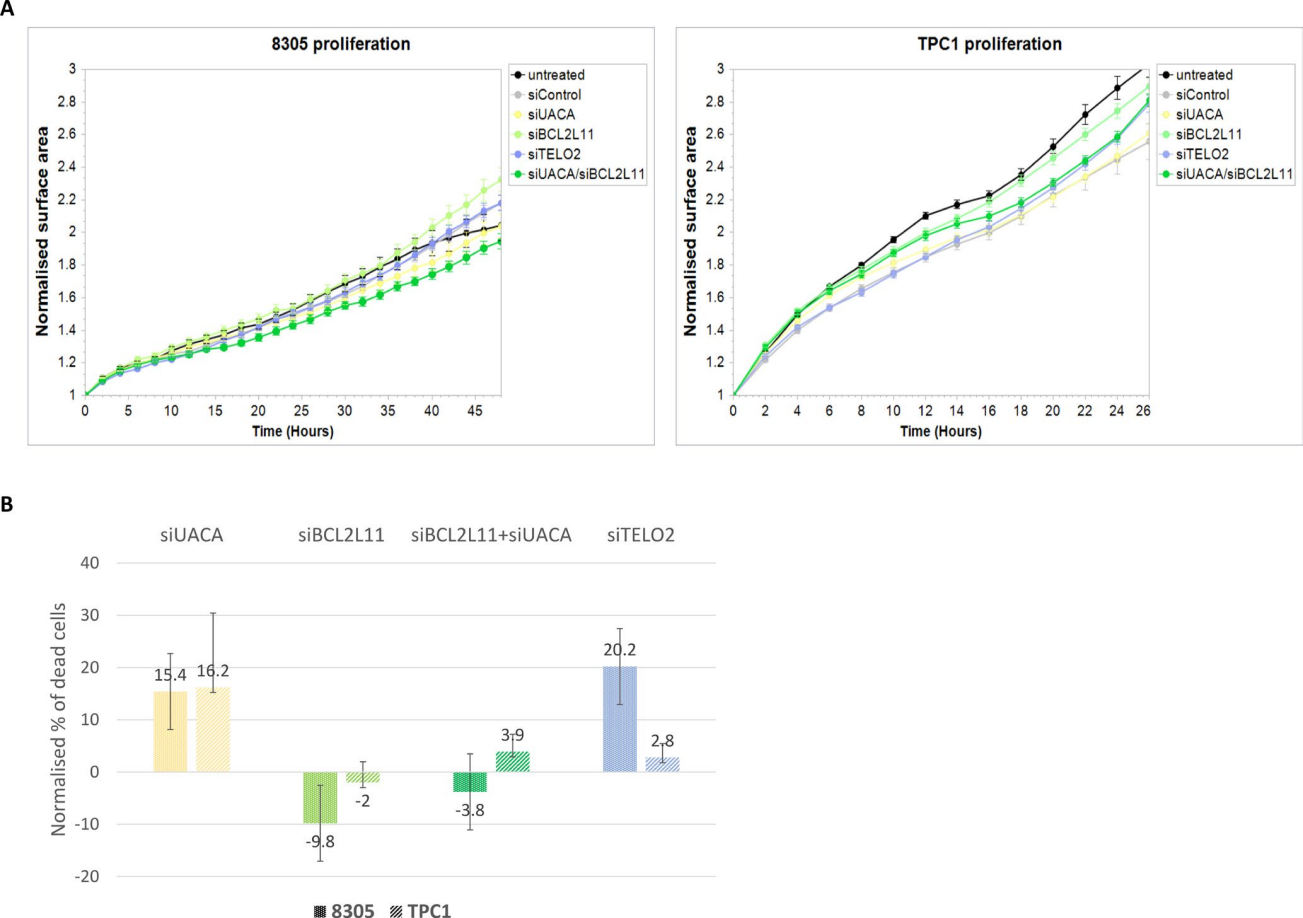
Proliferation and apoptosis analysis in 8305 and TPC1 cell lines following gene inhibition. (A) Incucyte proliferation analysis showed a mild proliferation increase in 8305 cells following *BCL2L11* inhibition relative to the control, while inhibition of other targeted genes did not affect proliferation. In contrast, no significant differences in proliferation rates were observed in the TPC1 cell line following any gene inhibition. (B) Flow cytometry analysis of dead cell ratios under cisplatin‐induced stress in both 8305 and TPC1 cell lines. Dead cell ratios were measured relative to siControl (siCont).

The inhibition of *UACA*, *BCL2L11*, or *TELO2* did not result in any significant changes in the proliferation of 8503 and TPC1 cell lines. Additionally, mild effects were observed with *BCL2L11* inhibition in the 8305 cell line. (Figure 4A).

### 3.6 UACA, BCL2L11, and TELO2 Effect on Apoptosis

Apoptosis was induced by application of cisplatin 48 h after transfection of siRNA, to coincide with the most efficient inhibition of candidate genes (Figure 3B–C).

After 2 days of cisplatin treatment, flow cytometry analysis was performed to evaluate the effect of gene inhibition on apoptosis (Figure 4B). The persistence of the inhibition was confirmed by RT‐PCR (data not shown).

Since it was difficult to distinguish necrosis from apoptosis in our experiment, cell populations considered only dead versus alive cells, and normalization was performed according to the number of living cells in the control group. Inhibitions of *UACA* and *TELO2* increased the number of dead cells compared to control siRNA, suggesting increased apoptosis in thyroid cancer cell lines. *TELO2* inhibition effects varied between cell lines, likely due to differing inhibition rates. BCL2L11 inhibition reduced dead cells, supporting its pro‐apoptotic role. Dual‐inhibition of *UACA* and *BCL2L11* reveals only a mitigated effect, consistent with the opposite roles of UACA and BCL2L11.

## 4 Discussion

WES analysis of 5 FMNTC families identified, in each family, around 10 rare (MAF < 1%) variants with a splice or truncating effect, which segregated with thyroid cancer cases. Although this efficient strategy targets rare, loss of function variants, it excludes activating variations in oncogenes. For each gene identified, we then examined its biological function, its expression in healthy thyroid tissue and in thyroid cancer, and its possible interaction with thyroid oncogene pathways. This enabled us to narrow down the list of genes of interest to three (*UACA*, *BCL2L11*, and *TELO2*) for which we carried out functional analyses.

It should be noted that, like other studies, the functional data were obtained by studying thyroid cancer cell lines. Inhibition of the various genes in these cell lines might have a lower oncogenic effect compared to healthy thyroid cells.

The splice‐site mutation NM_138621.4:c.498 + 3A>T; p.? identified in *BCL2L11*, shared by the grandmother, her son, and grandson who had thyroid cancer at ages 73, 45, and 17, is predicted to abolish the donor site of exon 8. Although no LOH was observed, RT‐PCR and immunohistochemistry on the tumour sample showed no expression of the gene product, unlike in the paired healthy tissue. Mechanisms other than LOH, such as promoter hypermethylation or somatic mutation, could induce this loss of expression of the second allele.

*BCL2L11* encodes a pro‐apoptotic protein that regulates intrinsic cell death in response to different stimuli. BCL2L11 can induce apoptosis directly by activating BAX and BAK secondary to JNK phosphorylation and indirectly by reducing interactions with some anti‐apoptotic proteins [15]. Several studies have reported that *BCL2L11* acts as a tumor‐suppressor gene in a variety of cancers, such as gastric cancer and rectal cancer [16, 17]. It has been shown that BCL2L11 inhibits migration and invasion while promoting apoptosis in gastric cancer, and it was recently published that silencing *BCL2L11* in a thyroid cancer cell line increases cell migration and invasion and restricts apoptosis [17, 18]. Our functional data confirm the effect of silencing *BCL2L11* on apoptosis in thyroid cancer cell lines.

The regulation of *BCL2L11* depends on two key proteins described in thyroid carcinogenesis: RUNX3 and FOXO3A [15]. FOXO3A is a forkhead transcription factor involved in the regulation of cellular homeostasis, modulating gene transcription of apoptosis, proliferation, cell cycle, survival, and response to DNA damage and oxidative stress. FOXO3A acts as a strong tumor suppressor in different cancers, including thyroid carcinoma. Sequestering FOXO3A in the cytoplasm leads to downregulation of *p27kip* and *BCL2L11* in thyroid carcinoma. It was suggested as a novel escape mechanism from p27kip‐induced cell cycle arrest and BCL2L11‐promoted apoptosis in thyroid cancer, hereby driving cancer cells from the inhibition of growth to cellular proliferation [19].

RUNX3 belongs to the RUNX family of transcription factors and shows strong tumor suppressor activity by modulating a series of cancer‐associated genes, such as p53, p21, Notch1, and p27, which are involved in the regulation of epithelial proliferation and apoptosis [20]. *BCL2L11* is another upregulated target of *RUNX3*. In thyroid cancers, the RUNX1 promoter is frequently inactivated by hypermethylation, and specific sites of methylation have been correlated with the risk of recurrence in PTC [21].

We also analysed a large control population cohort from unrelated noncancer individuals (gnomAD v.3.1.2: 76156 whole genomes). On the MANE transcript (NM_138621.5 which matches the transcript ENST00000393256), we found 32 high‐confidence predicted loss‐of‐function variants of *BCL2L11* in 53 gnomAD individuals, with a combined carrier frequency of 0.00003. The low frequency of truncating variants in the general population is therefore compatible with a gene involved in hereditary predisposition to cancer.

In summary, the rare variant c.498 + 3A>T of *BCL2L11* segregated with all the thyroid cases in this family, has a high predicted impact, no expression in the thyroid cancer sample, and is not reported in gnomAD version 4. Furthermore, *BCL2L11* is a known tumor suppressor gene in thyroid cancer cell lines, as shown by published functional studies and by our experiments. *BCL2L11* is therefore a good candidate gene to explain the hereditary predisposition to thyroid cancer in this family.

The NM_018003.3:c.3701del; p.(Ser1234Metfs*10) variant of *UACA* identified in three PTC patients from the same family (grandmother, son and grandson) causes a frameshift mutation resulting in a premature stop codon in exon 16. The loss of function of this mutation was confirmed by protein and gene expression analysis. Although no loss of heterozygosity (LOH) was observed, there was a complete loss of UACA protein expression in tumor cells, while adjacent normal cells retained their expression.

**FIGURE 5:**
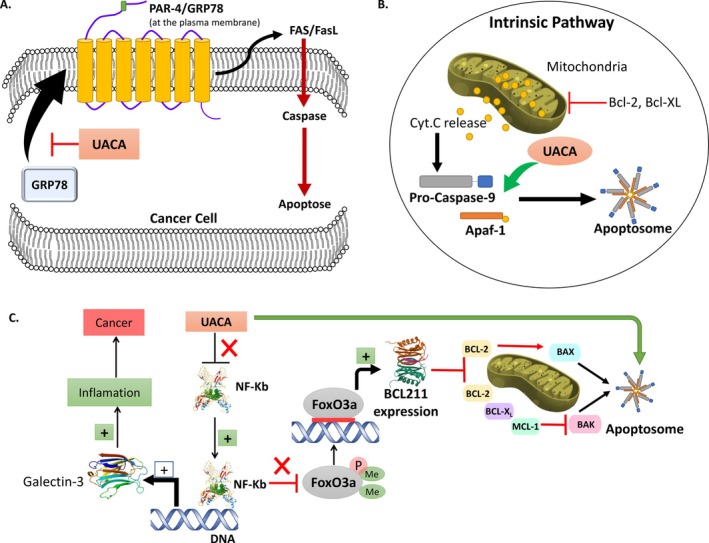
Apoptotic pathways of the candidate genes of hereditary predisposition to PTC. (A) UACA function in NF‐κB/Galectin‐3 pathway. FOXO3A activates the transcription of BCL2L11 pro‐apoptotic protein. UACA and BCL2L11 interact with the same apoptotic FOXO3A signalling pathway. (B) Pro‐apoptotic activity in the intrinsic pathway of UACA. (C) Anti‐apoptotic pathway of UACA.

UACA is proposed to act as a tumor suppressor by promoting apoptosome formation and inducing apoptosis in the intrinsic pathway during cellular stress. Indeed, UACA as a suppressor of the anti‐apoptotic protein NF‐κB (Figure 5B) [22] induces cytoplasmic retention of NF‐κB and thus its inactivation. Deficiency of UACA confers resistance to apoptosis secondary to upregulation of NF‐κB and Galectin‐3 [23]. Galectin‐3 is a lectin protein known to be involved in the malignant transformation, progression, and invasion of non‐medullary thyroid carcinoma [24, 25, 26].

Nevertheless, anti‐apoptotic activity has also been shown for UACA by sequestering PAR‐4. The PAR‐4/GRP78 pair translocates from the endoplasmic reticulum to the plasma membrane, leading to cell apoptosis. It has been suggested that suppression of UACA activity causes apoptosis by isolating extracellular PAR‐4 and preventing the transport of GRP78 to the cell surface, resulting in NF‐κB action (Figure 5C) [27]. This suggests that UACA has anti‐apoptotic activity by inhibiting the apoptotic protein PAR‐4.

Our functional analysis was consistent with an anti‐apoptotic effect (increased rate of apoptosis with SI RNA). There was no significant effect on proliferation. While the literature describes two opposing behaviors of *UACA*, our study did not support a direct tumor suppressor role for *UACA* in the apoptosis pathway in the thyroid cell line. However, such a role cannot be fully ruled out, as an increase in apoptosis could indirectly result from impaired DNA repair or other stress responses triggered by loss of tumor suppressor gene function. The involvement of UACA in these latter mechanisms has not been documented and was not investigated in this study.

In this study, we identified variations in both *BCL2L11* and *UACA* in affected individuals from the same family (A), leading to the hypothesis that these alterations may contribute to apoptosis dysregulation and oncogenesis. An oligogenic hypothesis was proposed, requiring concomitant inactivation of two genes in the concerned signaling pathways to induce a high risk of cancer. Double inhibition of *UACA* and *BCL2L11* in cancer cell lines did not show a significant effect on apoptosis or proliferation. Although healthy thyroid cells may behave differently, this seems to rule out this hypothesis.

The *TELO2* nonsense mutation NM_016111.3:c.1792C>T; p.(Gln598*), shared by 2 first degree relatives with thyroid cancer at ages 21 and 23, introduces a premature stop codon in exon 15, resulting in a loss‐of‐function allele. No LOH was observed and gene expression analysis could not be performed. However, protein expression analysis showed significant under‐expression in the nuclei of tumor cells, suggesting reduced activity.

TELO2 is a key regulator of phosphatidylinositol 3‐kinase‐related kinases (PIKKs), forming a complex with TTI1 and TTI2 and cooperating with Hsp90 to stabilize six PIKK proteins: ATM, ATR, DNA‐PKcs, SMG1, mTOR, and TRAPP16 [28]. ATM, ATR, and DNA‐PKcs play critical roles in DNA repair, cell cycle control, and apoptosis, with ATM and DNA‐PKcs involved in double‐strand break repair and ATR in single‐strand break repair [28, 29]. Loss of TELO2 function can impair these repair mechanisms. SMG1, involved in mRNA quality control, has been implicated in inflammation and cancer predisposition, while mTOR positively regulates cell proliferation [30]. In our functional analyses of thyroid cancer cell lines, we did not observe a tumor suppressor role for *TELO2* in the apoptotic pathway, as reducing *TELO2* expression resulted in increased apoptosis. Nevertheless, we were not able to test if TELO2 was implicated in the DNA repair pathway.

## 5 Conclusion

In conclusion, three genes of interest for hereditary predisposition to papillary thyroid cancer (PTC) have been identified in two of the five families by whole exome sequencing (WES). These genes are well expressed in normal thyroid tissue and underexpressed in tumor tissue. As reported in the literature, their functions are compatible with tumor suppressor activity, and loss of function/expression on tumor sample was confirmed for UACA and BCL2L11. The functional studies in our study showed that inhibition of *BCL2L11* increased cell proliferation mildly and reduced apoptosis, supporting its role in FNMTC. A case–control study should be carried out to test the association between *BCL2L11* mutations and FNMTC. However, the data from our cell culture experiments do not support a role of *UACA* and *TELO2* in proliferation or apoptosis in thyroid cancer. Their function in other carcinogenic pathways, such as DNA repair, warrants further investigation.

## Conflicts of Interest

The authors declare no conflicts of interest.

## Supporting information


**Data S1:** cge70060‐sup‐0001‐Supinfo.pptx.

## Data Availability

The data that support the findings of this study are available on request from the corresponding author. The data are not publicly available due to privacy or ethical restrictions.
