# Supplementary material for: Identification of BCL2L11 as a Candidate Gene for Hereditary Predisposition to Non‐Medullary Thyroid Cancer Using Familial Whole‐Exome‐Sequencing
Source: Clin Genet. 2025 Sep 3;109(3):458–69. doi: 10.1111/cge.70060 (PMC12881209; doi:10.1111/cge.70060)
Supplement: Supplementary file 1 — Data S1: cge70060‐sup‐0001‐Supinfo.pptx. [file CGE-109-458-s001.pptx]

## Slide 1
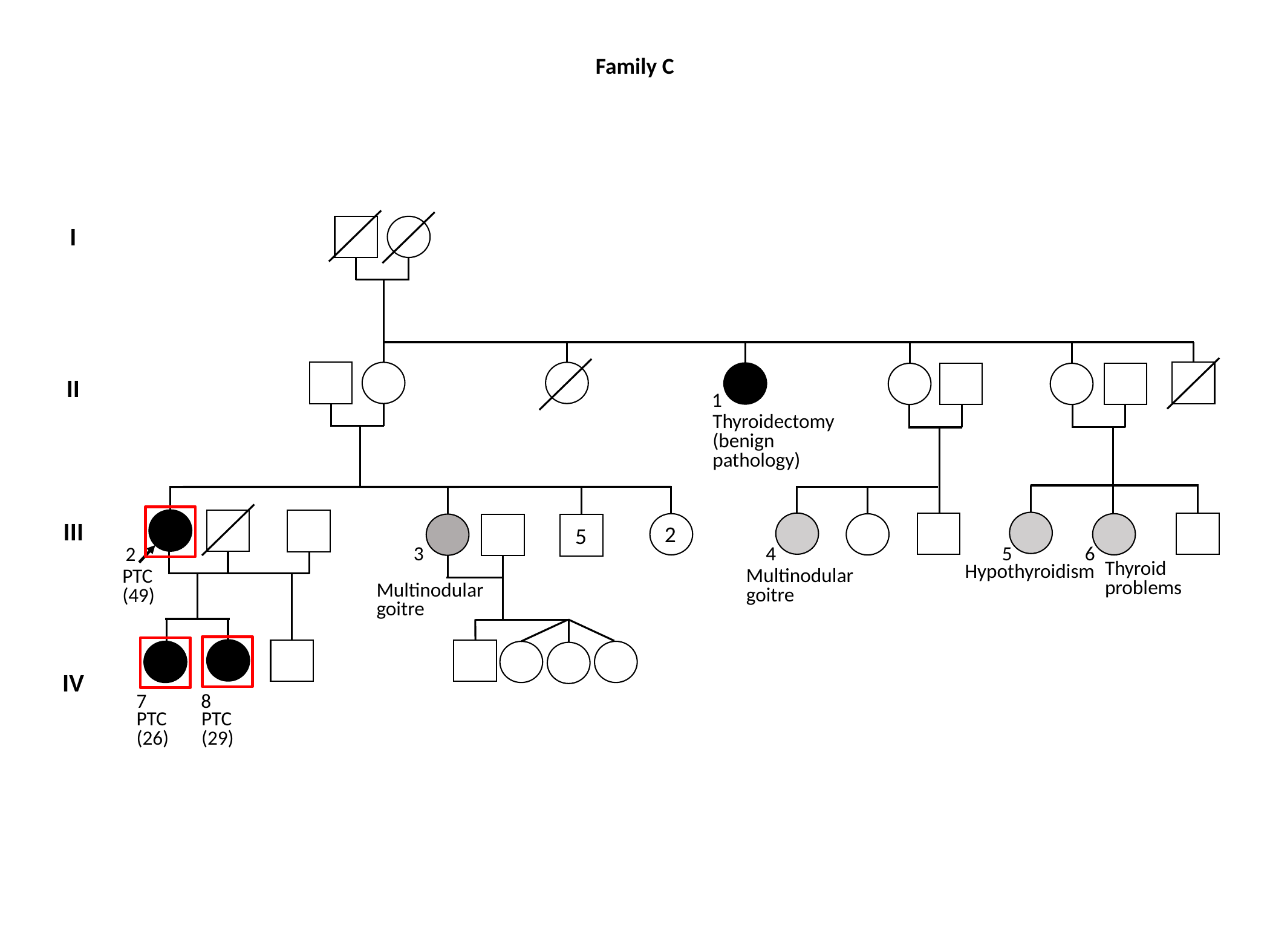

Family C
2
5
Hypothyroidism
Thyroid problems
Multinodular goitre
PTC
(49)
Multinodular goitre
PTC
(26)
PTC
(29)
1
Thyroidectomy (benign pathology)
6
3
4
5
2
7
8
I
II
III
IV

## Slide 2
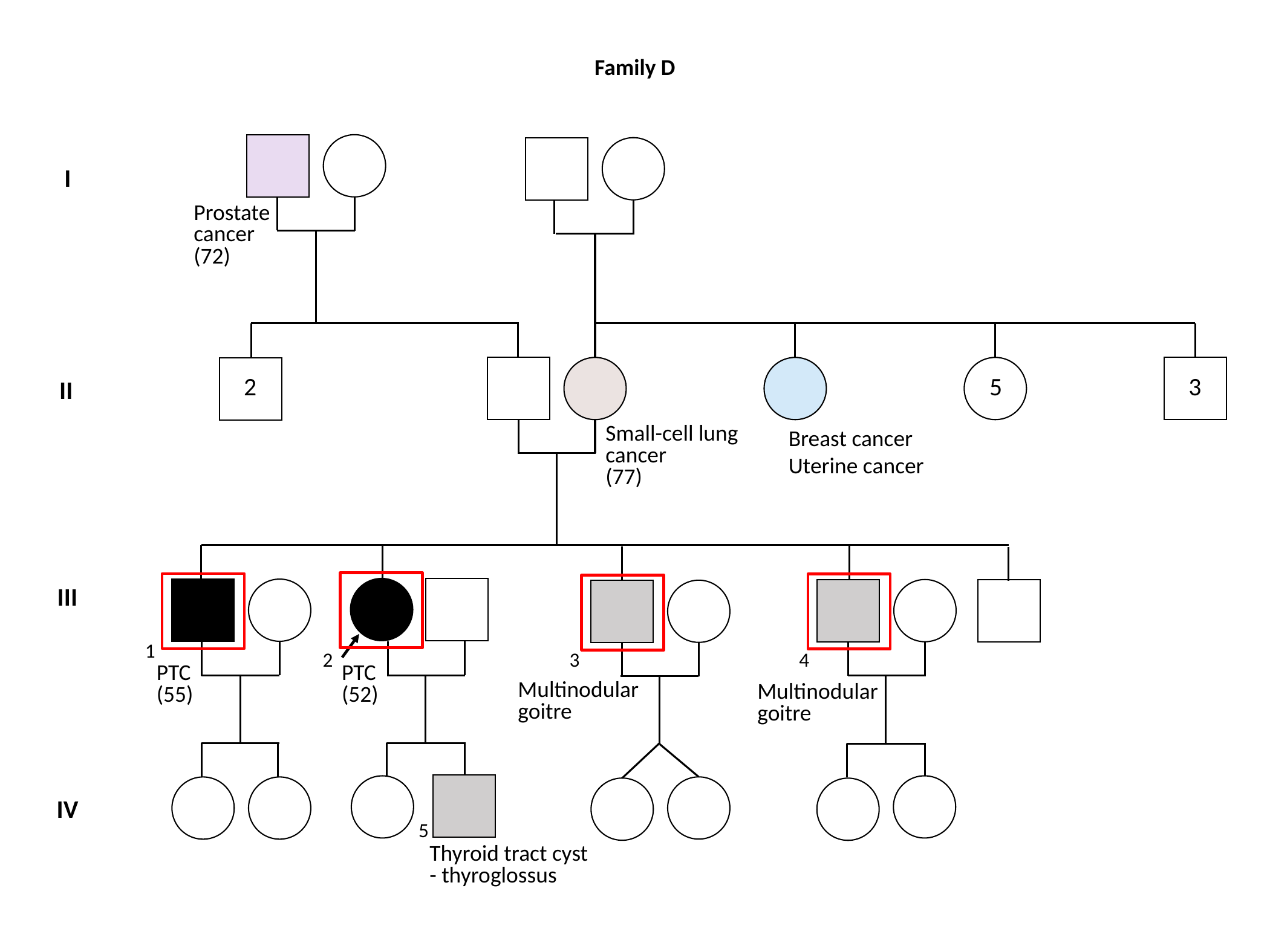

Family D
2
I
II
III
IV
Prostate cancer
(72)
5
3
Small-cell lung cancer
(77)
Breast cancer
Uterine cancer
1
2
3
4
PTC
(55)
PTC
(52)
Multinodular goitre
Multinodular goitre
5
Thyroid tract cyst - thyroglossus

## Slide 3
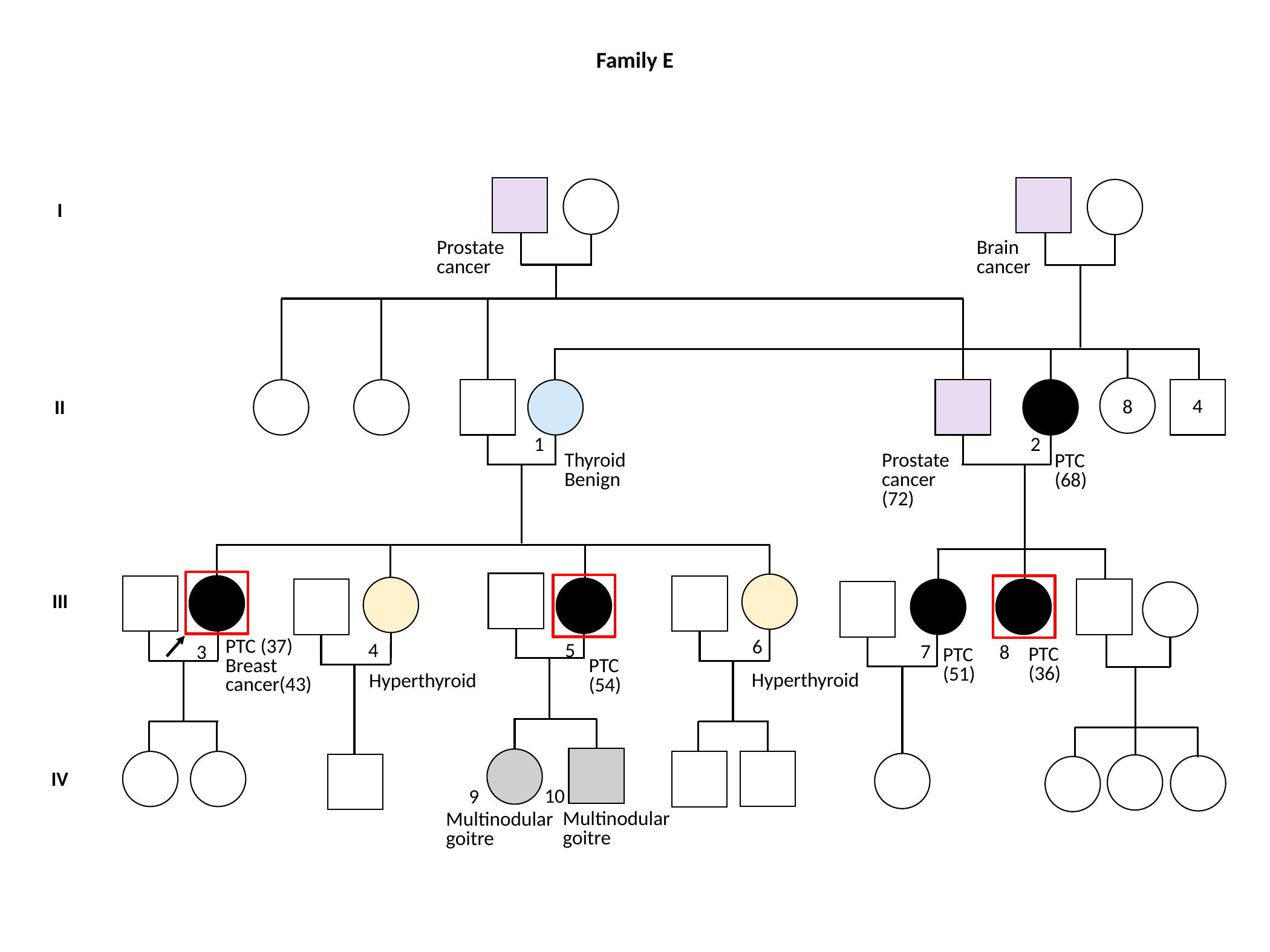

Family E
Prostate cancer
Brain cancer
Prostate cancer
(72)
PTC
(68)
PTC (37)
Breast cancer(43)
PTC
(36)
PTC
(51)
PTC
(54)
8
4
Thyroid Benign
Hyperthyroid
Multinodular goitre
Multinodular goitre
I
II
III
IV
6
7
1
2
5
4
8
3
Hyperthyroid
10
9
